# Association Analysis of the Extended MHC Region in Celiac Disease Implicates Multiple Independent Susceptibility Loci

**DOI:** 10.1371/journal.pone.0036926

**Published:** 2012-05-17

**Authors:** Richard Ahn, Yuan Chun Ding, Joseph Murray, Alessio Fasano, Peter H. R. Green, Susan L. Neuhausen, Chad Garner

**Affiliations:** 1 Department of Epidemiology, University of California Irvine, Irvine, California, United States of America; 2 Department of Population Sciences, Beckman Research Institute of City of Hope, Duarte, California, United States of America; 3 Department of Medicine and Immunology, The Mayo Clinic, Rochester, Minnesota, United States of America; 4 Center for Celiac Research, University of Maryland School of Medicine, Baltimore, Maryland, United States of America; 5 Celiac Disease Center, Columbia University, New York, New York, United States of America; Albert Einstein Institute for Research and Education, Brazil

## Abstract

Celiac disease is a common autoimmune disease caused by sensitivity to the dietary protein gluten. Forty loci have been implicated in the disease. All disease loci have been characterized as low-penetrance, with the exception of the high-risk genotypes in the *HLA-DQA1* and *HLA-DQB1* genes, which are necessary but not sufficient to cause the disease. The very strong effects from the known HLA loci and the genetically complex nature of the major histocompatibility complex (MHC) have precluded a thorough investigation of the region. The purpose of this study was to test the hypothesis that additional celiac disease loci exist within the extended MHC (xMHC). A set of 1898 SNPs was analyzed for association across the 7.6 Mb xMHC region in 1668 confirmed celiac disease cases and 517 unaffected controls. Conditional recursive partitioning was used to create an informative indicator of the known *HLA-DQA1* and *HLA-DQB1* high-risk genotypes that was included in the association analysis to account for their effects. A linkage disequilibrium-based grouping procedure was utilized to estimate the number of independent celiac disease loci present in the xMHC after accounting for the known effects. There was significant statistical evidence for four new independent celiac disease loci within the classic MHC region. This study is the first comprehensive association analysis of the xMHC in celiac disease that specifically accounts for the known HLA disease genotypes and the genetic complexity of the region.

## Introduction

Celiac disease (gluten-sensitive enteropathy, celiac sprue) is a common T cell auto-immune disease caused by sensitivity to the dietary protein gluten. It is primarily a disease of Caucasians, with a population prevalence of approximately 1% [1], with evidence suggesting that it is increasing in incidence [2], [3]. Co-occurrence of celiac disease with other autoimmune diseases has been noted, including type I diabetes, autoimmune thyroiditis, Sjögren's syndrome, Addison's disease, alopecia areata, inflammatory bowel disease, and adult rheumatoid arthritis [4], [5], [6], [7].

The role of histocompatibility antigens in the major histocompatibility complex (MHC) in celiac disease was first reported 30 years ago [8], [9] with the identification of HLA DQ2 almost 20 years ago [10]. Linkage studies to identify highly penetrant genes were conducted, but other than at HLA, no consistent high-risk loci were identified [11], [12], [13], [14], [15], [16], [17], [18]. More recently, genome-wide association studies (GWAS) and follow-up studies have identified 39 non-HLA loci that are associated with celiac disease, all together explaining approximately 5% of the disease risk [19], [20], [21], [22], [23]. In GWAS, the strongest associations by a large magnitude were to the MHC region, with the most strongly associated SNP explaining approximately 35% of the disease risk.

The HLA class II molecules, particularly the DQ2 molecule because of its unique peptide binding motif, are necessary for celiac disease and encode for cell surface proteins on CD4+ T lymphocytes that recognize gliadin [24]. The specific DQ molecule that is expressed, i.e., the serotype, is determined by the alleles present in the HLA-DQA1 and HLA-DQB1 HLA class II genes. Greater than 90% of celiac disease cases express HLA DQ2 [25], [26], [27], [28]. Approximately 5% of celiac disease cases are DQ8 [29], [30], [31], with the remaining 3–5% of celiac disease cases carrying neither DQ8 nor DQ2, although most carry a DQB1*02 allele. However, the HLA association is necessary but not sufficient for celiac disease to develop. Approximately 30% of the population carries the HLA DQ2 genotype, yet only 1% of the population develop the disease [32], [33], [34].

An investigation of the extended MHC (xMHC) for additional disease-associated common variants has not been conducted. Focused studies of the MHC in systemic lupus erythematosus (SLE) [35] and type 1 diabetes (IDDM) [36], [37], [38] have found evidence for novel, independent genetic associations in the region. While the specific statistical approaches utilized by these previous studies are unique, the studies shared a common approach. The known HLA disease alleles were characterized in an informative way then included in the association analysis to account for the known risk alleles while searching for new disease alleles. In this study, taking a similar approach, we test the hypothesis that there are additional disease-associated common variants in the HLA region other than the known *HLA-DQA1* and *HLA-DQB1* disease alleles.

## Results

**Figure 1:**
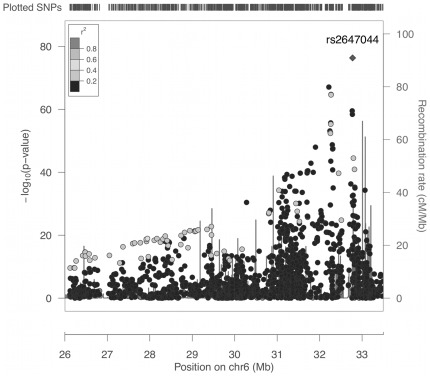
Association results for 1898 SNPs across xMHC, without accounting for the known HLA high-risk genotypes in the statistical analysis. Vertical bars indicate recombination rates generated from HapMap database. All pairwise linkage disequilibrium coefficients (*r* ^2^) included the most significantly associated SNP, rs2647044.

The 1898 SNPs spanning the xMHC were analyzed for association with celiac disease with a simple logistic regression model that included only the SNP genotype as the predictor of disease. The purpose of this analysis was to assess the associations between the 1898 SNPs and celiac disease without any statistical adjustment for the known high-risk genotypes at the *HLA-DQA1* and *HLA-DQB1* genes. The results of the association analysis are shown in Figure 1. The strongest association was found at rs2647044 with many other SNPs showing significant association near the *HLA-DQA1* and *HLA-DQB1* genes. SNP rs2647044 is approximately 35 kb centromeric of *HLA-DQB1* and 60 kb from *HLA-DQA1*. Analysis of pairwise LD, measured by the *r* ^2^ value, between rs2647044 and every other SNP showed that there were no other SNPs among those tested that were strongly correlated with the most significant SNP (Figure 1). The SNPs were expected to show low *r* ^2^ values because they were drawn from an Illumina GWAS platform and would have been selected for high informativity and low redundancy. Recombination hotspots are clearly apparent and delineate changes in the patterns of association between the SNPs and disease (Figure 1).

**Figure 2:**
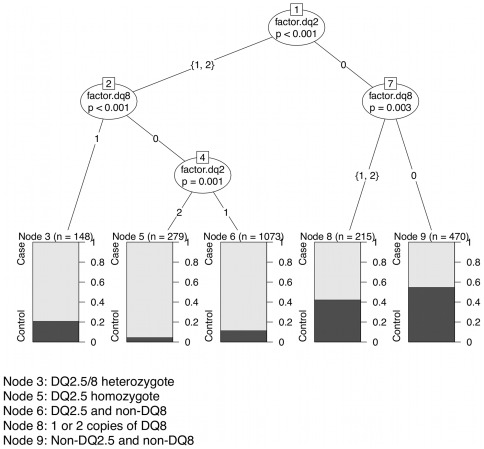
Binary tree computed by conditional recursive partitioning on HLA-DQA1 and HLA-DQB1 genotypes.

With very strong association originating from the known HLA high-risk genotypes it was impossible to identify independent SNP associations, hence we first utilized a statistical procedure to generate an informative categorical variable that marked the known genotype effects, and then we included this categorical variable in our logistic regression model. A categorical variable was constructed by conditional recursive partitioning to account for the HLA high-risk alleles. The common HLA genotypes associated with the highest risk of developing celiac disease are HLA-DQ2.5 and HLA-DQ8. The HLA-DQ2.2/7 genotypes also result in HLA-DQ2.5 through *in trans* combination of haplotypes. The *HLA-DQA1* and *HLA-DQB1* typing resulted in 16 possible multi-locus genotype categories. A conditional inference tree model based on the *HLA-DQA1* and *HLA-DQB1* genotypes was computed that resulted in five terminal nodes (Figure 2). As expected, the terminal nodes that were most informative of the outcome were the DQ2.5 homozygotes (n = 279 samples) followed by heterozygotes with one copy of DQ2.5 and one non-DQ8 haplotype (n = 1073), and the DQ2.5/DQ8 heterozygotes (n = 148 samples). Information dropped off significantly for the final two terminal nodes, with the worst case identification rate for those samples with non-DQ2.5 and non-DQ8 haplotypes (n = 470). The recursive partitioning algorithm could not further split the node representing samples with either 1 or 2 copies of the DQ8 haplotype (n = 215). The tree model showed no evidence that the effects of either the DQ2.5 or the DQ8 haplotypes were multiplicative. Table 1 shows the frequency distribution of the categorical variable created from the five terminal nodes of the recursive partitioning analysis. All binary splits resulting in the five terminal nodes were significant at p-values less than 0.003.

**Table 1:** Characteristics of Five-Level Variable for Known HLA High-Risk Alleles Computed by Conditional Recursive Partitioning.

Genotype	Cases	Controls	Total
DQ2.5/DQ8 heterozygote	117 (0.07)	31 (0.06)	148 (0.07)
DQ2.5 homozygote	266 (0.16)	13 (0.03)	279 (0.13)
DQ2.5/non-DQ8 heterozygote	949 (0.57)	124 (0.24)	1073 (0.49)
1 or 2 copies of DQ8	124 (0.07)	91 (0.18)	215 (0.10)
non-DQ2.5/non-DQ8	212 (0.13)	258 (0.50)	470 (0.22)
Total	1668 (1.00)	517 (1.00)	2185 (1.00)

**Figure 3:**
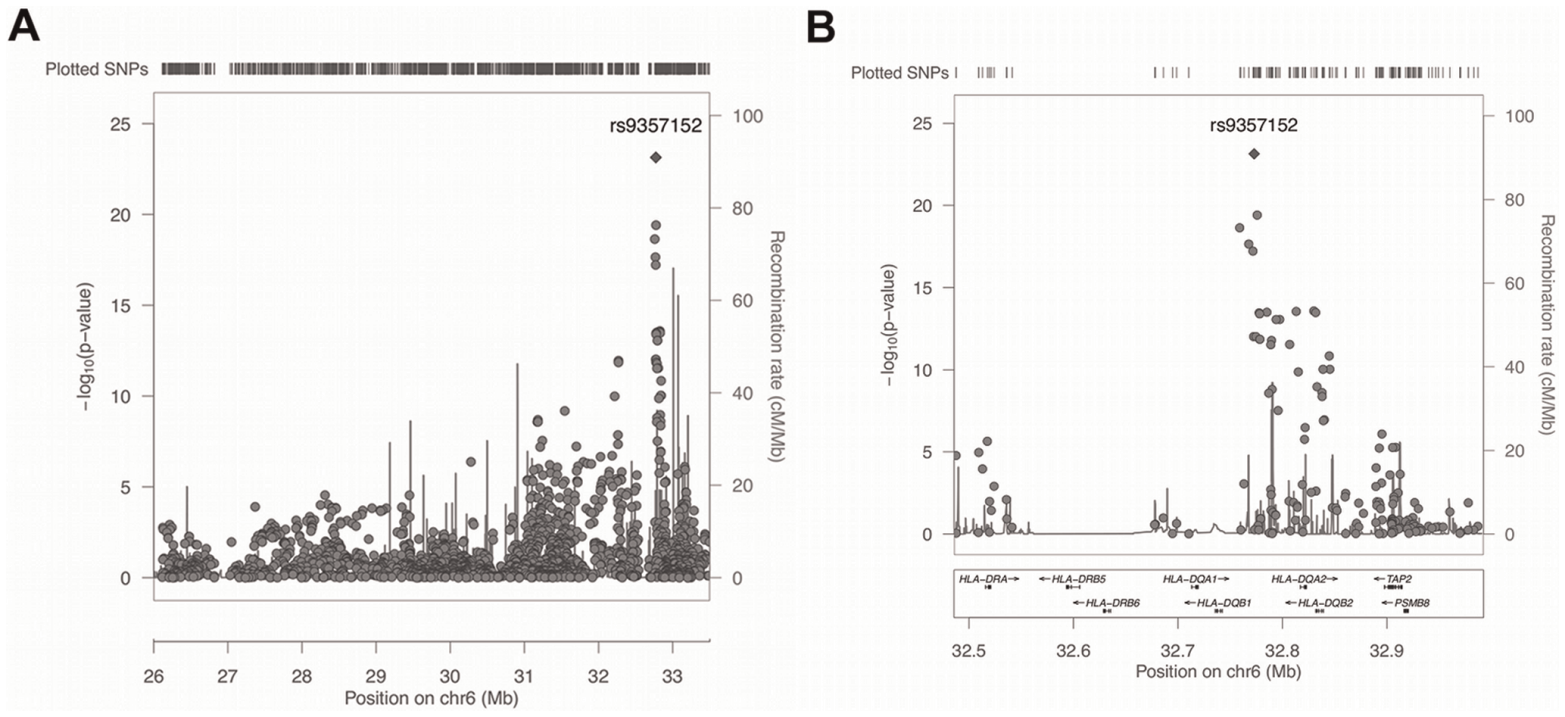
Association results for 1898 SNPs across (a) full xMHC, and (b) focused on the region around the known HLA class 2 celiac disease genes, accounting for known HLA high-risk genotypes in the statistical analysis. Vertical bars indicate recombination rates generated from HapMap database. All pairwise linkage disequilibrium coefficients (*r* ^2^) included the most significantly associated SNP, rs9357152.

The 1898 SNPs were each tested in a logistic regression model that included the categorical variable as well as SNP rs1063355. The SNP rs1063355 occurs in the 3′ untranslated region (UTR) of the *HLA-DQB1* gene and it was included in the association model because it is known to be strongly associated with the *HLA-DQB1* high-risk allele and was among the GWAS SNPs. In logistic regression model with rs1063355 as the only predictor of celiac disease, the SNP had a p-value of less than 1.0×10^−17^; however, adding the computed categorical variable to the model resulted in rs1063355 having a p-value of 2.5×10^−5^, indicating relatively weaker but significant residual effects on the disease outcome. The purpose of including rs1063355 in the association model was to eliminate the possibility of the SNP being identified as an independent predictor of the disease, since it is already known, and to account for any residual effects from *HLA-DQB1* not identified by the categorical variable. Figure 3a shows the results of the association analysis, and demonstrate the impact of the adjustment for the known HLA high-risk genotypes. While rs2647044 (*P* = 3.85×10^−20^) was still highly significant, rs9357152 (*P* = 7.28×10^−24^) became the most significantly associated SNP when the effects from the known HLA high-risk genotypes were accounted for in the logistic regression model. The SNP rs2647044 is approximately 3.3 kb from rs935152. In Figure 3b, focused on the *HLA-DQA1* and *HLA-DQB2* intergenic region, several SNPs remained significantly associated within the region of the known celiac *DQA1* and *DQB1* disease genes.

A SNP grouping method was carried out to identify a minimal set of SNPs with independent effects on the disease across the xMHC. This analysis used results from the association analysis that accounted for the known *HLA-DQA1* and *HLA-DQB1* high-risk genotype effects. Independent sets of SNPs were constructed according to their correlated effects on the disease outcome, with each set being identified by a single index SNP that was most informative of the group and most strongly associated with disease. Grouping was based on the following parameters: the significance threshold for index SNPs of a p-value less than 5×10^−7^; a secondary significance for grouped SNPs of a p-value less than 0.01; a maximum physical distance of 250 kb over which the SNPs in the group can span; and an LD threshold, measured by *r^2^*, of 0.1 or greater with the index SNP. The parameters were chosen to be conservative such that the validity of the results was maximized.

**Figure 4:**
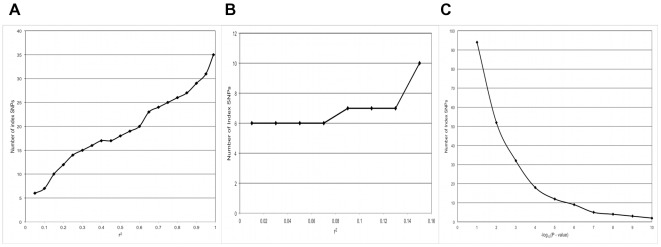
Results of sensitivity analysis for SNP grouping analysis showing the relationship between the group linkage disequilibrium parameter (*r* ^2^) and the number of index SNPs identified, with *r* ^2^ ranging from (a) 0.05 to 0.95, and focused on the range from (b) 0.01 to 0.15. Results (c) show the relationship between the minimum statistical significance parameter for the association between the disease and the index SNP and the number of index SNPs identified.

An analysis was carried out to determine the relationships between the parameter values used in the SNP grouping procedure and the number of SNPs identified. As described in the Methods section, the grouping procedure depends on several user-defined input parameters. The purpose of this analysis was to determine the sensitivity of the method to the input parameter values so the values used in the subsequent grouping analysis were not arbitrarily set. The analysis showed that the number of index SNPs identified largely depended on the significance threshold for the index SNP and the LD between the index SNP and the secondary SNPs within each group. In Figure 4a, the linear relationship between the numbers of index SNPs identified and the *r* ^2^ parameter, with *r* ^2^ values ranging from 0.05 to 1.0 are shown. As secondary SNPs are required to show greater *r* ^2^ values with the index SNP, more groups containing fewer SNPs are formed, resulting in more index SNPs. The relationship between the number of index SNPs and the *r* ^2^ values between 0.01 and 0.15 is shown in Figure 4b, where the number of index SNPs reaches a minimum of six at an *r* ^2^ value of 0.07. The *r* ^2^ value was set at 0.10 as a tradeoff between the ability to distinguish independent associations, while accepting that long-range LD is predominant across the MHC and weak correlations between independently acting loci are likely. The relationship between the significance threshold for the index SNP [measured as −1×log_10_ (p-value for association)] and the number of index SNPs identified is shown in Figure 4c. The number of index SNPs showed an exponential decline as the index SNP statistical significance threshold increased [i.e., the p-value decreases and −1×log_10_ (p-value) increased], with the parameter showing a declining impact on the number of index SNPs at a p-value of 1.0×10^−6^ [−1×log_10_ (p-value) = 6]. The number of index SNPs will minimize at zero when the threshold exceeds the statistical significance of all SNPs in the experiment. The p-value threshold of 5.0×10^−7^ [−1×log_10_ (p-value) = 7] was selected to be below the point at which the parameter has a large effect on the number of index SNPs identified but large enough that the procedure could select from many putative independent loci. The analysis determined that the secondary significance level and the physical distance parameters had weak effects on the number of index SNPs identified (results not shown).

**Figure 5:**
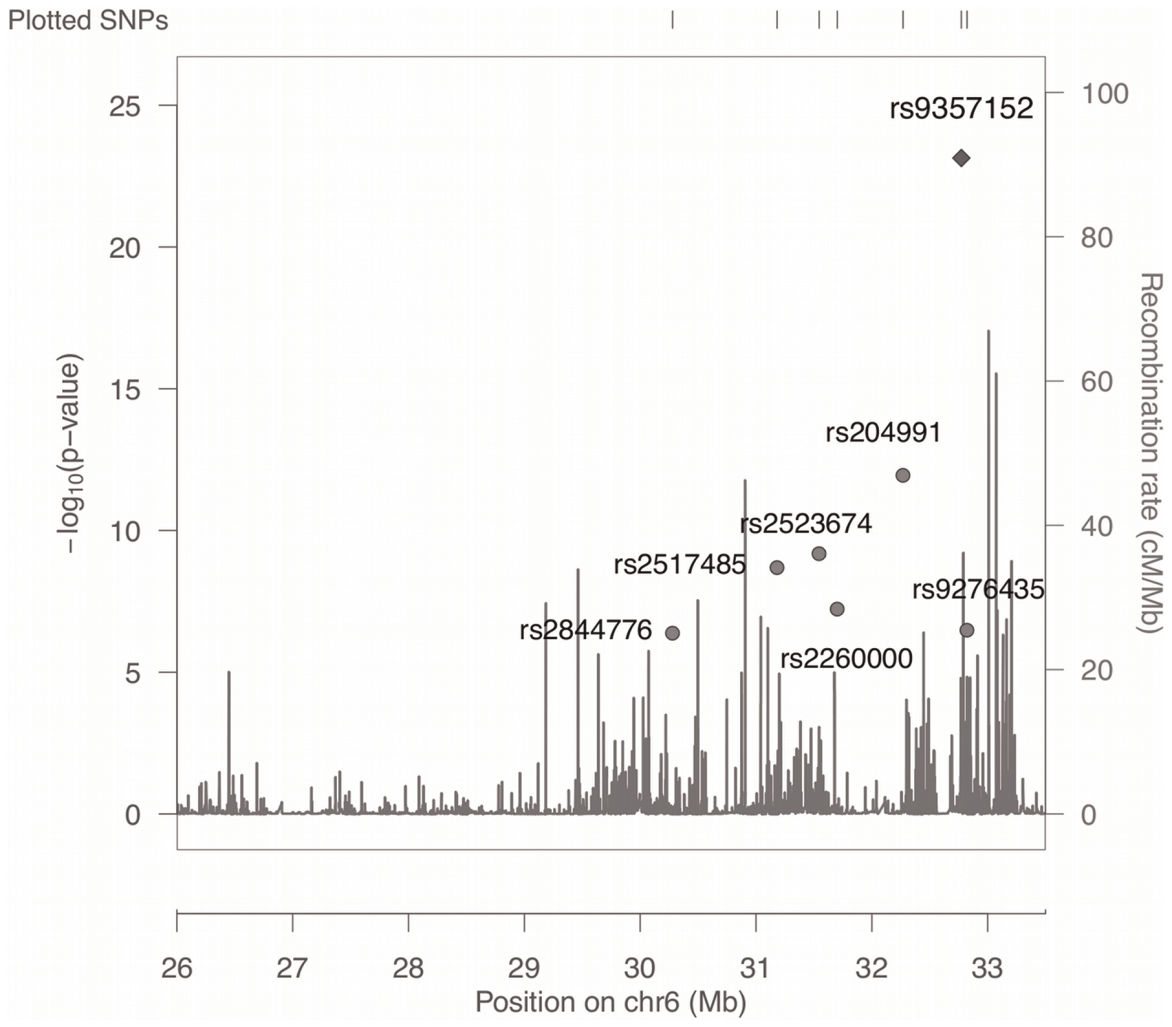
Association analysis results and locations of seven index SNPs identified by grouping analysis of the xMHC. Recombination rates were estimated from HapMap data and are indicated by vertical bars.

The grouping procedure resulted in the identification of seven index SNPs that showed independent effects on the celiac disease outcome, in addition to the known HLA high-risk types that were accounted for in the analysis. The positions of the seven loci across the xMHC as well as the estimated rates of recombination are shown in Figure 5. The seven loci all map within the classical MHC region and are separated by hotspots of recombination. In table 2, the seven index SNPs are ranked by their statistical significance, with all seven showing p-values less than the defined index SNP significance threshold of 5.0×10^−7^. Four of the SNPs had odds ratios greater than 1.0 indicating that the minor allele was at increased frequency among cases, while the remaining three SNPs showed the opposite effect and the more frequent allele was predominant among cases. The top four most statistically significant index SNPs (rs937152, rs204991, rs2523674 and rs2517485) each tagged over 30 secondary SNPs with a combined total of 135, while the remaining three index SNPs (rs2260000, rs9276435 and rs2844776) tagged a combined total of 34 secondary SNPs (Table 2).

**Table 2:** Association Results for Seven Index SNPs Representing Independent Loci.

SNP	Position	Minor allele	Minor allele freq.	P-value	Odds ratio (95% C.I.)	No. Secondary SNPs	Multiple LR P-value No HRA Adj.#	Multiple LR P-value HRA Adj	Functional Genes*
rs9357152	32664960	G	0.12	7.28×10^−24^	0.24 (0.21–0.28)	34	0.0004	0.00012	HLA-DQB1
rs204991	32161366	G	0.46	1.13×10^−12^	2.24 (2.00–2.51)	33	3.31×10^−9^	0.0024	GPSM3
rs2523674	31436789	A	0.35	6.55×10^−10^	0.57 (0.52–0.62)	33	0.0014	0.00693	HCP5
rs2517485	31074101	A	0.49	2.03×10^−9^	1.77 (1.61–1.95)	35	6.53×10^−5^	0.0022	SEEK1/PSORS1C1
rs2260000	31593476	G	0.22	5.90×10^−8^	0.60 (0.54–0.66)	15	0.23	0.39	BAT2
rs9276435	32713867	A	0.40	3.28×10^−7^	1.82 (1.62–2.05)	5	0.00014	0.10	HLA-DQA2
rs2844776	30171827	G	0.37	4.17×10^−7^	1.65 (1.50–1.83)	4	0.052	0.096	TRIM26

* Either the gene that the SNP occurs in, or the nearest gene within the same LD haplotype block as the index SNP.

# Indicates adjustment for known common high-risk alleles by inclusion of the five-level variable computed by recursive partitioning.

To further verify the independence of the seven index SNPs, they were analyzed simultaneously for association in two multiple logistic regression models. In the first model, the seven SNPs were the only predictors of celiac disease. Table 2 shows that five of the seven index SNPs remain statistically significant with p-values<0.01 when they are modeled together. SNPs rs2260000 (p-value = 0.23) and rs2844776 (p-value = 0.052) were not significant predictors of the disease when analyzed simultaneously with the other five SNPs. The second multiple logistic regression model included the seven index SNPs and the five-level categorical variable for the known common HLA high-risk genotypes that was computed by recursive partitioning. This analysis identified four of the seven SNPs as being significant predictors of celiac disease including, rs9357152 (p-value = 0.00012), rs204991 (p-value = 0.0024), rs2523674 (p-value = 0.00693) and rs2517485 (p-value = 0.0022). Each of the four SNPs tagged more than thirty other SNPs across the MHC region. The multiple logistic regression analysis results showed that rs9357152, rs204991, rs2523674 and rs2517485 were statistically significant independent markers of new celiac disease loci within the MCH.

## Discussion

GWAS have successfully identified 39 non-HLA loci showing signficant association with celiac disease, with modest predictive information [19], [23]. Alleles of the *HLA-DQA1* and *HLA-DQB1* genes are necessary for celiac disease but are not sufficient for disease development. The xMHC contains more than 250 expressed genes of which many are involved in immune-regulation [39]. However, it had not been thoroughly interrogated for additional celiac disease loci due to the complicated nature of the analysis, including the very strong effects of the known disease alleles, the extraordinary genetic variation within the region, and the complex patterns of linkage disequilbrium. A simple association analysis of the region that does not take these complications into account would generate misleading results (as seen in our comparison of simple and multiple logistic regression models). Statistical evidence was presented for four new and independent celiac disease susceptibility loci within the classical MHC. An informative measure of the known high-risk HLA genotypes was computed by recursive partitioning and coded as a categorical variable. The variable was included in an association analysis of the 7.6 Mb xMHC region using a set of 1898 SNPs that passed rigorous quality control assessments. The conditional recursive partitioning approach is superior to a sample stratification to account for the known HLA high-risk types; there is not a loss of power from sub-sampling, and it generates the most informative measure of the known effect. The independent associations were identified using a conservative grouping procedure that was designed to minimize the probability of false positive results.

The recursive partitioning analysis generated a variable that captured the effects of the known common *HLA-DQA*1 and *HLA-DQB1* high-risk genotypes on the disease outcome. Including the variable in the association analysis had a pronounced effect on the association results. Of the 671 SNPs that had p-values of less than 5.0×10^−7^ without including the high-risk genotype effects, 48 had p-values less than the threshold when the known HLA effects were accounted for in the analysis model. While the adjustment for the effects was effective, it is unlikely that it was complete. There are low frequency risk alleles and genotypes at *HLA-DQA1* and *HLA-DQB1* that were not specifically identified by the categorical variable because they were not common enough to be strong predictors of disease in the full sample. Furthermore, the complex genetic structure of the MHC is not completely amenable to straightforward statistical adjustment. Although residual influence from *HLA-DQA1* and *HLA-DQB1* high-risk genotypes is likely to exist, it is unlikely that the four independent disease loci identified in the analysis are due to unaccounted for correlations with the known HLA risk genotypes given the conservative approach that was taken in the study. The results strongly suggest additional celiac disease alleles are present in the MHC.

None of the SNPs reported in Table 2 occur in the coding sequence of genes or have known or reported functional effects. In Table 2, we list the functional genes that the SNPs occur within or the closest gene that is within the same LD haplotype block. SNP rs9357152, previously reported in a GWAS to be associated with celiac disease [22], is on the same haplotype block as rs9469220, which is associated with Crohn's disease [40], and rs6457617, which is associated with rheumatoid arthritis [41]. *HLA-DQB1* is the closest gene to rs9357152 at approximately 45 kb centromeric, however moderate recombination separates the index SNP from the gene. SNP rs204991 is in the third intron of the G-Protein Signaling Modulator 3 gene (*GPSM3*), within a haplotype block that encompasses the entire gene. HLA Complex P5 (*HCP5*) is the nearest gene to rs2523674 at about 4 kb, no other genes are within 20 kb of the SNP. Another SNP in *HCP5* was found to be significantly associated with severe cutaneous adverse drug reactions in a Japanese GWAS [42]. SNP rs2517485 is about 10 kb from the putative psoriasis and systemic sclerosis disease susceptibility gene (*SEEK1* or *PSORS1C1*) and the Corneodesmosin Precursor gene (*CDSN*).

The goal of this analysis was to test the hypothesis that the known *HLA-DQA1* and *HLA-DQB1* celiac disease high-risk alleles were not the only celiac disease alleles within the xMHC. The results show evidence for additional celiac disease loci within the 3.7 Mb classic MHC region, however no evidence was found for additional disease loci within the additional 4.1 Mb of the extended region. The index SNPs, rs9357152, rs204991, rs2523674 and rs2517485, were the strongest markers for the four new loci among the 1898 SNPs included in the analysis. Additional investigation is required to validate the reported findings and to locate the new disease alleles, including determining if these SNPs are causal, possibly playing roles in regulation of these genes, or if they are only tagging causal variants.

## Materials and Methods

### Study subjects

As part of our GWAS study, we formed a North American Celiac Disease Genetic consortium comprised of Dr. S. Neuhausen at COH and Dr. C. Garner at UC Irvine, Dr. J. Murray at the Mayo Clinic, Dr. A. Fasano at the University of Maryland, and Dr. P. Green at Columbia University to study subjects enrolled in previous studies at each center. The already collected, coded, non-identifiable samples were contributed from City of Hope, Mayo Clinic, University of Maryland and Columbia University. The City of Hope Institutional Review Board approval was granted in January 2010, and the approved protocol is 09169. The Mayo Clinic participants were previously enrolled under the direction of Dr. Joseph Murray under an approved IRB protocol 1173-99. Similarly, the samples from the University of Maryland were collected under the direction of Dr. Alessio Fasano under three approved IRB protocols (H- 27784, H29090, and H-29938). Similarly, the samples from Columbia University were collected under the direction of Dr. Peter Green under approved IRB protocol AAAE8893 (and previous protocol #8562). All participants provided written informed consent as described in each of the protocols.

Reflecting the population of celiac disease, all 2300 participants were Caucasian. Of the celiac disease cases, 532 were from COH, 743 from the Mayo Clinic, 423 from the University of Maryland, and 66 from Columbia University. Of the controls, 177 were from COH and 359 from the Mayo Clinic, for a total of 1764 cases and 536 controls. Blood samples were collected for serology testing for celiac disease and DNA was extracted for genetic studies. The four sites used the same serological tests, collected similar questionnaire data, and used the same criteria for diagnosis of celiac disease. To be defined as celiac disease, each case was required to have: 1) positive celiac-specific autoantibody (IgA EMA and IgA tTG antibodies); 2) or a proximal small intestinal biopsy compatible with celiac disease; and 3) either clinical and/or histological improvement with a gluten-free diet. The majority of cases fulfilled all three criteria. A small proportion of subjects, predominantly those diagnosed before modern serology came into use, did not have celiac-specific serology, and another small minority of subjects did not have a biopsy. Those who tested positive for both IgA tTG and IgA EMA were considered positive for celiac disease. Using this sequential testing technique, sensitivity and specificity rates of virtually 100% were reported, making it practical to accurately identify those with celiac disease on the basis of serology alone [43], [44]. A small biopsy was performed on approximately 90% of those who tested positive serologically, and all had a positive biopsy for the disease. For all sites, individuals who were self-declared celiacs because of a self administered gluten-free diet and without a biopsy, or individuals whose biopsy demonstrated only minor changes such as increased intraepithelial lymphocytes and/or crypt hyperplasia, were not included as cases. The unaffected controls tested serologically negative for celiac disease.

### HLA typing

The process for determining the *HLA-DQA1* and *DQB1* alleles in the cases and controls first involved determining the genotypes of 95 of the individuals by Sanger sequencing. This set of 95 individuals acted as positive controls for the HLA genotyping of the remaining case and control individuals. To identify controls for genotyping for each of the *HLA-DQA1* and *DQB1* alleles, we directly sequenced the second exons of DQA1 and DQB1 for 95 samples by Sanger sequencing using the ABI Prism BigDye Terminator cycle sequencing kit 3.1 (PE Applied Biosystems). Sequences were aligned using Sequencher software (GeneCode Corporation, MI), as well as manually inspected if needed. DQ allele assignments were done manually by comparing variants from each sample to variants of each DQ allele downloaded from dbMHC sequence alignment viewer (http://www.ncbi.nlm.nih.gov/gv/mhc). Using those samples with DQ alleles determined by direct sequencing, *HLA*-*DQA1* and *DQB1* genotypes were determined by one of two high-throughput DQ typing methods. Three hundred and eighty nine samples (from COH) were genotyped by an allele-specific PCR method developed in our laboratory by Feolo et al. [45]. Genotyping accuracy for this method is greater than 98%. The remaining 1816 samples were genotyped by a tagging SNPs approach in which six tagging SNPs were used to predict four different HLA DQ types (DQ2.5, DQ2.2, DQ7 and DQ8) associated with celiac disease [46]. Genotyping call rates ranged from 95% to 99% and duplicate concordance rates were higher than 99%. There was 100% concordance between the 95 HLA genotypes determined by Sanger sequencing and the same individuals' HLA genotypes imputed from the six tagging SNPs.

### Genotype data

A sample of 2300 individuals plus duplicates were genotyped at the Center for Inherited Disease Research (CIDR) using the Illumina 660 W Quad GWAS platform. Individuals and SNPs with less than 98% complete GWAS genotype data were excluded from the sample. SNPs with a minor allele frequency less than 0.03 or failing a test of Hardy-Weinberg equilibrium with a p-value less than 1.0×10^−5^ were excluded. GWAS genotype data were used to test if data were consistent with second degree or higher familial relationships and the reported sex, and inconsistencies that could not be resolved resulted in sample exclusion. Analysis for population stratification and admixture using multidemensional scaling and cluster analysis revealed a predominant single cluster and resulted in the exclusion of several apparent ancestral outliers. A sample of 2185 individuals, including 1668 confirmed celiac disease cases and 517 unaffected controls, passed all QC criteria, had complete HLA typing information, and were used for association analysis in the current study. There were 1898 SNPs between positions 26,000,508 and 33,544,122 on chromsome 6p, encompassing the xMHC that were part of the Illumina GWAS panel, that passed all QC assessments and were used for the current association analysis.

### Conditional inference based recursive partitioning

A conditional inference-based recursive partitioning method was used to partition the sample of individuals into strata based on their *HLA-DQA1* and *HLA-DQB1* genotypes in such a way as to minimize the within-group heterogeneity. The recursive partitioning method is a two-stage process in which predictor variables are selected and then the sample is subjected to a binary split [47]. A global test of independence between all the input variables and the outcome was carried out to select independent predictor variables. If the null hypothesis of independence could be rejected at a pre-determined p-value threshold of 0.05, the input variable with the strongest association to the response was selected. The extent of association was measured by the p-value corresponding to a test of the partial null hypothesis of a single input variable and the response variable. A binary split was then imposed on the selected input variable. These steps were repeated until the global null hypothesis of independence could be rejected. A factor variable was created from the terminal nodes of the tree resulting from the binary splitting process. The terminal nodes define the set of strata that each individual is assigned to based on their *HLA-DQA1* and *HLA-DQB1* genotypes. The conditional inference based recursive partitioning was computed using the PARTY package in R [48].

### Association analysis

Association analysis was performed by fitting logistic regression models to the observed disease status and genotype data, with the celiac disease outcome predicted by the SNP genotype. The unphased genotypes 1/1, 1/2 and 2/2 were coded as 0, 1 or 2 to denote the number of minor alleles present. *HLA-DQA1* and *HLA-DQB1* effects on disease risk were accounted for by incorporating the multi-level factor variable computed by recursive partitioning into a multiple regression model with the SNP genotype. The simple and multivariate regression models were computed using PLINK and the GenABEL package in R [49], [50].

### Linkage disequilibrium (LD)-based SNP grouping

A data reduction procedure was used to group sets of SNPs that had highly correlated associations with the celiac disease outcome and to determine how many SNPs showing significant association in the adjusted logistic regression were likely to be independent. SNPs were grouped together into LD groups (or ‘clumps’) by rank orders of p-values and decreasing LD, measured by *r* ^2^, from the top or ‘index’ SNP in each group. In addition to an LD threshold within each group, non-index SNPs also had to meet a specified secondary p-value threshold and be within a physical distance threshold (kb) from the index SNP. The LD-based SNP grouping analysis was computed using the ‘clump’ procedure in PLINK [50].
